# Influence of Various Light Regimes on Morphofunctional Condition of Transplantable Melanoma B16

**DOI:** 10.3390/biomedicines11041135

**Published:** 2023-04-10

**Authors:** David A. Areshidze, Maria A. Kozlova, Maxim V. Mnikhovich, Tatyana V. Bezuglova, Valery P. Chernikov, Zarina V. Gioeva, Aleksey V. Borisov

**Affiliations:** Avtsyn Research Institute of Human Morphology of Federal State Budgetary Scientific Institution “Petrovsky National Research Center of Surgery”, 117418 Moscow, Russia

**Keywords:** melanoma, light pollution, melatonin, tumor growth suppression, circadian rhythm

## Abstract

A study of the morphofunctional condition of mice with transplantable melanoma B16 under the influence of a normal daylight regime, constant lighting and constant darkness was conducted. It was shown that exposure to constant lighting leads to intensification of the proliferation of melanoma cells, more significant growth and spread of the tumor, the development of more pronounced secondary changes, the presence of perivascular growth and an increase in perineural invasion. At the same time, keeping of animals in constant darkness significantly reduced the intensity of the proliferative process in the tumor and lead to tumor regression in the absence of signs of lympho-, intravascular and intraneural invasion. Intergroup differences in tumor cell status were confirmed by the results of micromorphometric studies. It was also shown that the expression of clock genes was suppressed by an exposure to constant light, while an influence of constant darkness, on contrary, led to its intensification.

## 1. Introduction

Biological systems of all levels of organization are characterized by the rhythm of functioning processes, which is one of the fundamental properties of living matter. For various biological systems, rhythms with different periodicity are described: from fractions of a second to dozens of years. Circadian rhythms are one of the most significant types of biorhythms for mammals [1]. The set of CR biological processes in various organ systems forms a strictly coordinated ensemble, which is the chronostructure of the organism. The presence of this organized rhythmic structure of biological processes provides the necessary order for their flow and makes it possible to maintain the functioning of body systems at an optimal level [2], the violation of the circadian rhythm leads to the development of desynchronosis, which provokes the occurrence of pathologies [3].

The circadian structure of the body is a hierarchically organized complex and includes various structures, but a special place in the organization of the chronostructure of mammals belongs to the interaction of the suprachiasmatic nuclei of the hypothalamus with the pineal gland. The pineal gland acts as the most important circadian pacemaker and at the same time is the leading link for the implementation of circadian signals. The production of melatonin, the main hormone of pineal gland, shows clear daily periodism: production begins with the onset of the dark time of the day, reaches maximum values at midnight and is stopped by exposure to light [4].

At the molecular-genetic level, the biological clock includes the clock genes *Bmal*, *Clock*, the *Per* gene family and the *Cry* genes, as well as some other genes whose activity affects the above-mentioned key genes, or which are controlled by them [5,6,7,8,9].

Nighttime exposure to light, often called “light pollution”, has increased significantly and become an essential part of modern lifestyles, with consequences in the form of many severe disruptions in the behavior and health of individuals, including oncologic pathologies. Light exposure at dark times of day, which results in disturbances in endogenous circadian rhythmicity and suppresses nocturnal melatonin secretion in the pineal gland, is now considered a significant factor which could cause an increase in the number of developing neoplasms [10,11].

The number of studies showed an influence of night darkness deprivation on spontaneous tumorogenesis in some mammals [12,13,14,15]. It is well-known that constant lighting also has an activating effect for the development of chemically induced carcinogenesis in laboratory animals [16,17]. Likewise, an influence of constant lighting promotes the growth of transplanted tumors [18,19].

Melatonin, a hormone synthesized during the dark period mainly by the pineal gland of all vertebrates including mammals, which acts as an adapting signal to the light–dark cycle, is currently considered as a broad-spectrum adaptogen. Its antioxidant, antiaromatase, antiestrogen, direct intracellular anticarcinogenic and antimetastatic properties, the ability to stimulate antitumor immunity, participation in the regulation of oncogenes and suppressor genes, inhibitory properties in relation to tumorogenesis, metastasing and neoangiogenesis are described [20,21,22,23,24,25,26].

Skin melanoma is one of the most common malignant neoplasms with a dangerous and unpredictable clinical course. The rapid increase in the incidence of this disease is a global trend. The tumor is characterized by rapid growth and early formation of metastases spreading by lymphatic and hematogenous routes. Producing very wide spectrum of biologically active substances such as pituitary and hypothalamic hormones, enkephalins and catecholamines, melanomas affect homeostasis of an organism significantly to benefit the tumor at the expense of the host [27].

At the same time, the question of the dual role of melanin pigmentation of melanoma in the pathological process is very complicated. On the one hand, melanin pigment plays a crucial role in the skin protection against the injurious effects of environmental stressors such as ultraviolet radiation and other factors, and protects an organism against the development of different types of skin cancer; on the other hand, its presence may also be necessary for the malignant transformation of melanocytes. It is shown that melanogenesis by itself and its highly reactive intermediates show some cytotoxic, genotoxic, and mutagenic activities which can lead to melanoma progression and resistance to immunotherapy [28].

There are numerous pieces of evidence that melatonin deficiency and a violation of the structure of the circadian rhythms of an organism as a result of it are one of the factors causing the development of this pathology [29,30].

The MT1 receptor is widely distributed in most cells of mammalian skin in norm and pathology, including the keratinocytes, melanocytes, fibroblasts, squamous cell carcinoma and melanoma cells. MT2 receptors were also found both in normal and malignant melanocytes [31,32]. Melatonin is also a known to be a ligand for a retinoid-related orphan nuclear hormone receptor RZR/RORα. In addition, many physiological effects of melatonin are apparently associated with its interaction with intracellular proteins (calmodulin, calreticulin and tubulin), as well as with the fact that melatonin prevents the binding of Ca2+ to calmodulin [33]. It is described by a number of authors that loss of melatonin or its receptors due to dark deprivation leads to the development of pronounced oxidative stress in the skin, which may lead to an increase in the content of melanin in the cells of the skin and melanoma of the animals in conditions of constant lighting, since melanin exhibits antioxidant effects by scavenging free radicals, and may promote melanoma development and progression [34,35,36].

It is known that mammalian skin cells, including melanocytes, and melanoma cells do not only possess specific melatonin receptors, but are also a source of extrapineal melatonin themselves since it is synthesized and metabolized in them [37]. The content of extrapineal melatonin in the cells of both skin and melanoma increases during dark deprivation, but this increase is not sufficient to compensate for the deficiency in pineal melatonin [38]. It is also shown that this hormone inhibits melanoma growth in rodents and human due to its inhibitory effects on melanoma cell proliferation [39,40,41].

Melatonin has important antitumor actions, among which the antiproliferative effect stands out. Melatonin exerts an antiproliferative, oncostatic and anti-migrating effect on the melanoma model by interfering with the cytoskeleton organization, and also due to its inhibition of linoleic acid absorption [42,43,44,45].

The aim of our study was to investigate the influence of deprivation of darkness and light on parameters of growth intensity, morphological and immunohistochemical features of transplanted melanoma B16.

## 2. Materials and Methods

### 2.1. Experimental Objects

Eight-week-old male BDF1 hybrid mice (*n* = 75) of 21–22 g body weight, taken from USF «Vivarium and Animal Housing Group of Screening and Preclinical Studies Unit of Federal Research Center of Problem of Chemical Physics and Medicinal Chemistry RAS», were used in our study. A number of researchers consider BDF1 mice to be more suitable for studies of melatonin-mediated effects of light exposure on the organism in health and disease than C57BL6 mice, since the latter are characterized by defective melatonin production both in the pineal gland and in peripheral organs, including the skin [46,47].

Standard laboratory keeping of animals and all experimental exposures were made in concordance with the European Convention for the Protection of Vertebrate Animals used for Experimental and other Scientific Purposes (Strasbourg, 18 March 1986). The series of studies to which this work belongs was approved by the Bioethical Committee of the Federal State Budgetary Scientific Institution “Research Institute of Human Morphology”, proc No. 34 (10) (14 March 2022).

### 2.2. Study Design

Three equal groups of animals were used in the study.

Animals of the control group (*n* = 25) (control) were kept in conditions of a fixed light:dark regime using artificial lighting with fluorescent lamps (10:14 h, with light turned on at 8:00 and turned off at 18:00) (LD).

I experimental group (*n* = 25) underwent an influence of constant lighting (LL).

II experimental group (*n* = 25) underwent an influence of constant darkness (DD).

In accordance with the Russian sanitary standards for working premises lighting, light intensity per cage area unit made up 150 lux.

B16/F10 melanoma cell culture samples were obtained from N.N. Blokhin Russian Cancer Research Center (Moscow) and transplanted in the Experimental Tumor Chemotherapy Group of the Department of Kinetics of Chemical and Biological Processes, Institute of Problems of Chemical Physics of Russian Academy of Sciences (Chernogolovka). 0.5 mL samples of a suspension of tumor tissue of B16/F10 melanoma in medium 199 at a dilution of 1:10 by weight were subcutaneously injected to each animal in standard procedure, with use a 1.2 × 40 needle, into the area of the left flank closer to the back, after diethyl ether anesthesia [48,49,50,51,52].

A palpable tumor was detected on day 6 in animals exposed to constant illumination, and on day 7–8 in other groups.

Animal sacrifice by the method of cervical dislocation was made on the 15th day after melanoma transplantation with use of circadian time points at 9:00, 15:00, 21:00 and 03:00. Evisceration was carried out after euthanasia.

The measurements of tumor length, width and height were made, and the mass of animals and tumors was determined. Tumor volume was calculated by formula: V = π/6 × D1 × D2 × D3, where D1—length, D2—width, and D3—height of the tumor in centimeters.

### 2.3. Morphological and Morphometric Methods

Melanoma samples fixed in 10% neutral buffered formalin and underwent standard histological processing with final pouring into Histomix histological medium (BioVitrum, St. Petersburg, Russia) and preparation of serial sections of 5–6 μm thickness on sliding microtome Leica SM2010 R (Wetzlar, Germany). Staining of sections with hematoxylin and eosin was made in accordance with the standard histological technique [53]. Stained sections were fixed in BioMount mounting medium (BioVitrum, Russia).

Microscopic examination of melanoma preparations was made with use of Leica DM 2500 microscope and Leica DFC 290 digital camera (Germany). Karyo- and cytometry were carried out on 10 digital images of randomly selected visual fields taken from each studied preparation at a magnification of ×200, ×400 and ×1000. Fiji software package with appropriate plugins, a program built on the basis of ImageJ v2, was used for the implementation of morphometric studies [54]. Measurements of the cross-sectional area of the nuclei of cell (area of nuclei, Sn) and the cross-sectional area of the cell (area of cell, Scell) were carried out in micrometers after preliminary geometric calibration on an object-micrometer scale. Nuclear–cytoplasmic ratio of melanoma cells was calculated by the following formula: NCR = Sn/Sc, where: Sn is an area of nucleus of cell; Sc is an area of cytoplasm [55].

To determine the mitotic index, the number of mitotic figures and total count of melanoma cells per 1 mm^2^ cells was counted, results are represented as ppm (‰) [56].

### 2.4. Immunohistochemical Methods

For the setting of immunohistochemical tests, dewaxed melanoma sections were rehydrated and underwent 3% hydrogen peroxide solution treatment which blocked endogenous peroxidase activity. After unmasking of antigens by boiling the slides in citrate buffer (pH 6.0), the samples were placed into the «Ultra V Block» (Thermo Fisher Scientific, Waltham, MA, USA) solution; then the reactions with primary antibodies were performed [57]. Reactions with use of phosphate buffer solution instead of primary antibodies with served as controls.

List of used antibodies:

- *PER2*–Rabbit polyclonal (Cloud-Clone Corp., Houston, TX, USA), 1:200;
- *BMAL1*–Rabbit polyclonal (Cloud-Clone Corp., Houston, TX, USA), 1:200;
- *Clock*–Rabbit polyclonal (Cloud-Clone Corp., Houston, TX, USA), 1:200.

After processing, the sections were dehydrated in alcohols of ascending concentration and xylene in accordance with standard scheme and fixed with BioMount mounting medium (BioVitrum, St Petersburg, Russia).

The results of the immunohistochemical reaction were evaluated by the proportion of stained cells or cell nuclei (depending on the localization of the antigen) in relation to the total number of cells. Two investigators independently reviewed and evaluated samples, counting the number of cells showing characteristic staining. The percentage of stained cells was assessed in 4 fields of view at ×400 magnification. The expression of the studied genes was assessed by counting the percentage of positive cells of the total number of cells on each slide and expressed as a percentage of positive cells (0–100%) [58].

### 2.5. Measurement of Serum Melatonin Concentration

Blood samples were collected 4 times per day at 9:00, 15:00, 21:00 and 3:00. After centrifugation of samples at 3000× *g* for 15 min, the concentration of melatonin in blood serum was measured with use of commercial enzyme-linked immunosorbent assay (ELISA) kit (USCN Life Science Inc., Wuhan, China) in accordance with the manufacturer’s protocol. Melatonin concentrations of all of the samples were measured with an ELISA reader (Sunrise-Basic-Tecan, Grödig, Austria) set to an optical density at 450 nm and a standard range of 12.35–1000 pg/L.

### 2.6. Methods of Statistical Processing

Collected data were analyzed with use of “GraphPad Prism 6.0” software through calculation of the mean values, standard deviation, and standard error of the mean. Statistical differences were determined by the Kruskal–Wallis test. Differences were accepted as statistically significant at *p* < 0.05. The study presents average daily values from the parameters determined at 4 time points during the day.

## 3. Results

### 3.1. Influence of Various Light Regimes on Body Weight, Mass and Volume of the Tumor

**Figure 1:**
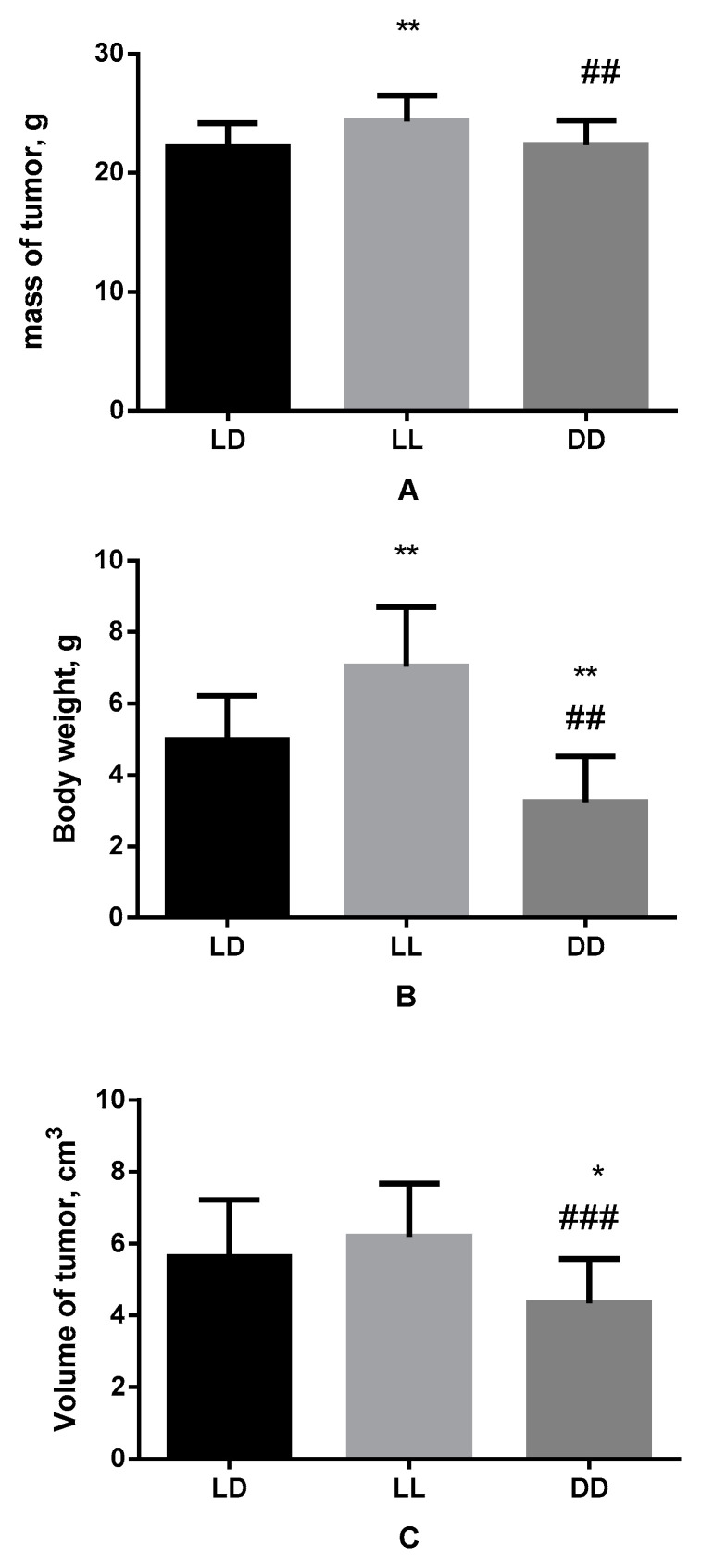
Influence of various light regimes on body weight of mice (**A**), mass of tumor (**B**) and its volume (**C**). Hereinafter: * (*p* ≤ 0.05); ** (*p* ≤ 0.005)—differences in comparison with the parameters of control group mice; ## (*p* ≤ 0.005); ### (*p* ≤ 0.0005)—differences in comparison with mice of LL group.

The results of the study show mouse body weight increased significantly under condition of darkness deprivation, making up 24.31 ± 0.41 g against of 22.16 ± 0.44 g in the control group. Herewith, the mass of the tumor also turned out to be higher, rising from 4.98 ± 0.27 g in the control group to 7.04 ± 0.31 g in animals kept under constant light. However, the volume of the tumor did not change significantly (Figure 1).

In the case of the influence of constant darkness there were no reliable changes in mouse body weight in comparison with the control; however, amounting to 22.35 ± 0.45 g, it was less than in mice kept under constant illumination (Figure 1).

The mass of the tumor as a result of keeping of mice in constant darkness was 3.25 ± 0.28 g, which is less than in both groups; the same is true for its volume, which was equal to 4.35 ± 0.27 cm^3^.

### 3.2. Influence of Various Light Regimes on Morphological Characteristics of B16 Melanoma

There were no macroscopic differences in the tumor between the groups, except of size. The tumors were represented by nodules of soft-elastic consistency, with heterogeneous dark brown or blackish coloration; the section showed a variegated appearance with focal necroses in some places.

**Figure 2:**
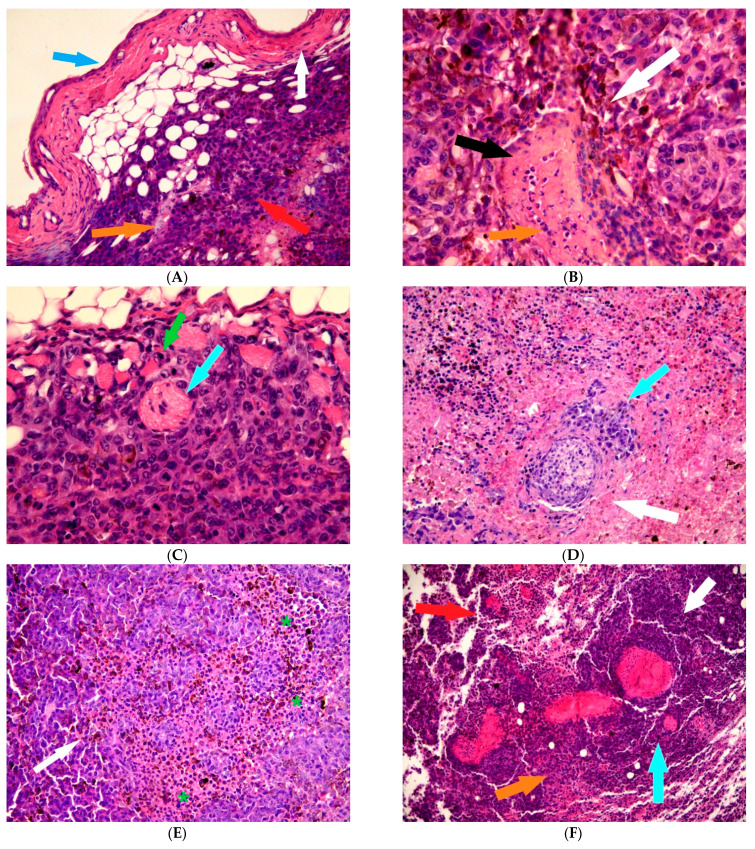

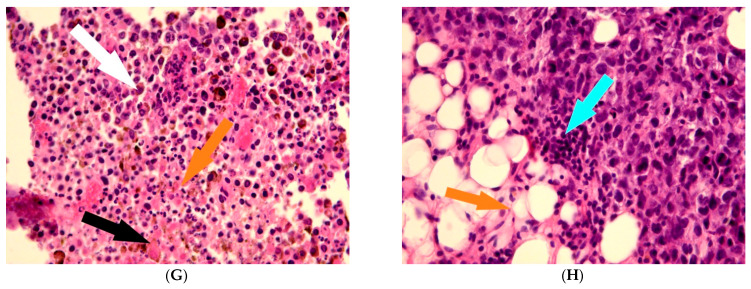
Mice melanoma, hematoxylin and eosin staining. (**A**)—LD group, ×200, the blue arrow shows hypoplasia of the stratified squamous epithelium with signs of acanthosis; white arrow—anastomosing acanthomatous strands at the border of the epidermal-dermal junction; red arrow—proliferation of atypical melanocytes; orange arrow—infiltration of all layers of surrounding tissues with tumor cells. (**B**)—LD group, ×400, white arrow—overgrowth of fields of tumor cells around a blood vessel with the formation of a rosette-like structure; black arrow—necrosis of vessel wall; orange arrow—areas of lymphocytic and neutrophil infiltration. (**C**)—LD group, ×400, light blue arrow shows areas of perineural invasion, green arrow indicates a mitotic figure. (**D**)—LL group, ×100, light blue arrow shows perineural invasion, white arrow—tumor cells reinforcing the area of the perineurium. (**E**)—LL group, ×100, white arrow shows epithelioid cells, asterisks show proliferating cell fields. (**F**)—DD group, ×100, white arrow—residual part of tumor in a form of overgrowth of epithelioid cell melanoma; light blue—plethoric sinusoidal type vessels; orange arrow—hemorrhages with severe lymphoplasmacytic infiltration, an admixture of eosinophils and an abundance of segmented leukocytes; red arrow—cells in condition of mitosis, foci of necrosis. (**G**)—DD group, ×400, regressive part of the tumor, white arrow shows lymphoplasmacytic infiltration; black arrow—infiltration with an admixture of eosinophils and an abundance of segmented leukocytes; orange arrow—site of micronecrosis with hemosiderin deposit. (**H**)—DD group, ×400, residual part of tumor, light blue arrow shows TILs; orange arrow—moderate infiltrate, focal fibrosclerosis of stroma.

Microscopic examination of the tumor in the LD group revealed that it consists of fields of proliferating atypical cells containing a large amount of melanin. Pigment content in the tumor was mainly intracellular; however, there were areas where it was located outside the cytoplasm. Epithelioid cells were the predominant population of tumor cells; multiple mitotic figures were noted (Figure 2).

The tumor had some signs of secondary changes represented predominantly by fields of necrosis, and decay, in a less degree. Perineural invasion of tumor was noted between areas of its necrobiosis—therefore, it possessed neurotorpism. Epithelioid tumor cells did not just surround the nerve trunks as a dense ring, but also an infiltration of perineurium with tumor cells complexes was noted. TILs (tumor infiltrating lymphocytes) infiltrate was moderate, with focal distribution and medium density (Figure 2).

Melanoma samples of animals of the LL group differed histologically from the control. The tumor consisted of fields of epithelioid cell melanoma containing a large amount of melanin. Localization of melatonin was predominantly intracellular. Epithelioid cells were mainly located in the vertical phase of growth, and there were also noted some areas of perivascular growth, but with absence of lympho- and intravascular invasion. Vascular walls were predominantly characterized with secondary changes, focal necrosis and edema. Multiple mitotic figures were noted in a tumor. Melanoma had clear signs of secondary changes, represented by fields of necrosis with areas of lymphocytic and neutrophil infiltration. There were areas of perineural invasion of tumor with the presence of invasion of tumor cells in the perineurium (intraneural invasion). TILs infiltrate presence was moderate, with focal distribution. Density of TILs infiltrate was also moderate. The skin in the projection of tumor growth was characterized by hypoplasia of stratified squamous epithelium with acanthosis phenomena, with anastomosing acanthomatous strands at the border of the epidermal–dermal junction and infiltration of all layers of the skin and surrounding soft tissues with melanoma cells.

Keeping of animals under conditions of constant darkness changed the structure of a tumor in an even greater extent, but the character of changes differed from LL group. This group showed signs of tumor regression. The residual part of a tumor was presented as overgrowths of epithelioid cell melanoma containing a large amount of melanin, which was located mainly intracellular. Plethoric vessels of the sinusoidal type, hemorrhages, pronounced lymphoplasmacytic infiltration with an admixture of eosinophils and an abundance of segmented leukocytes, high mitotic activity and foci of necrosis were noted. Epithelioid cells, as in the previous group, were in the vertical phase of growth, but without signs of lympho-, intravascular and perineural invasion. Moderate TILs infiltration and focal fibrosclerosis of stroma were noted in the residual part of the tumor.

In the regressive part of the tumor, single dystrophically altered tumor cells and also severe lymphoplasmacytic infiltration with an admixture of eosinophils and an abundance of segmented leukocytes, hemosiderin deposits and extensive areas of necrosis were noted.

Mitotic index made up 7.28 ± 0.54‰ in control group, in animals of LL group it increased significantly, reaching 10.05 ± 0.78‰, and in mice of DD group it decreased significantly to 6.03 ± 0.44‰.

### 3.3. Influence of Various Lighting Regimes on Micromorphometric Parameters of Cells of B16 Melanoma

We established the presence of reliable differences in micromorphometric parameters of B16 melanoma cells depending on light regimes.

**Figure 3:**
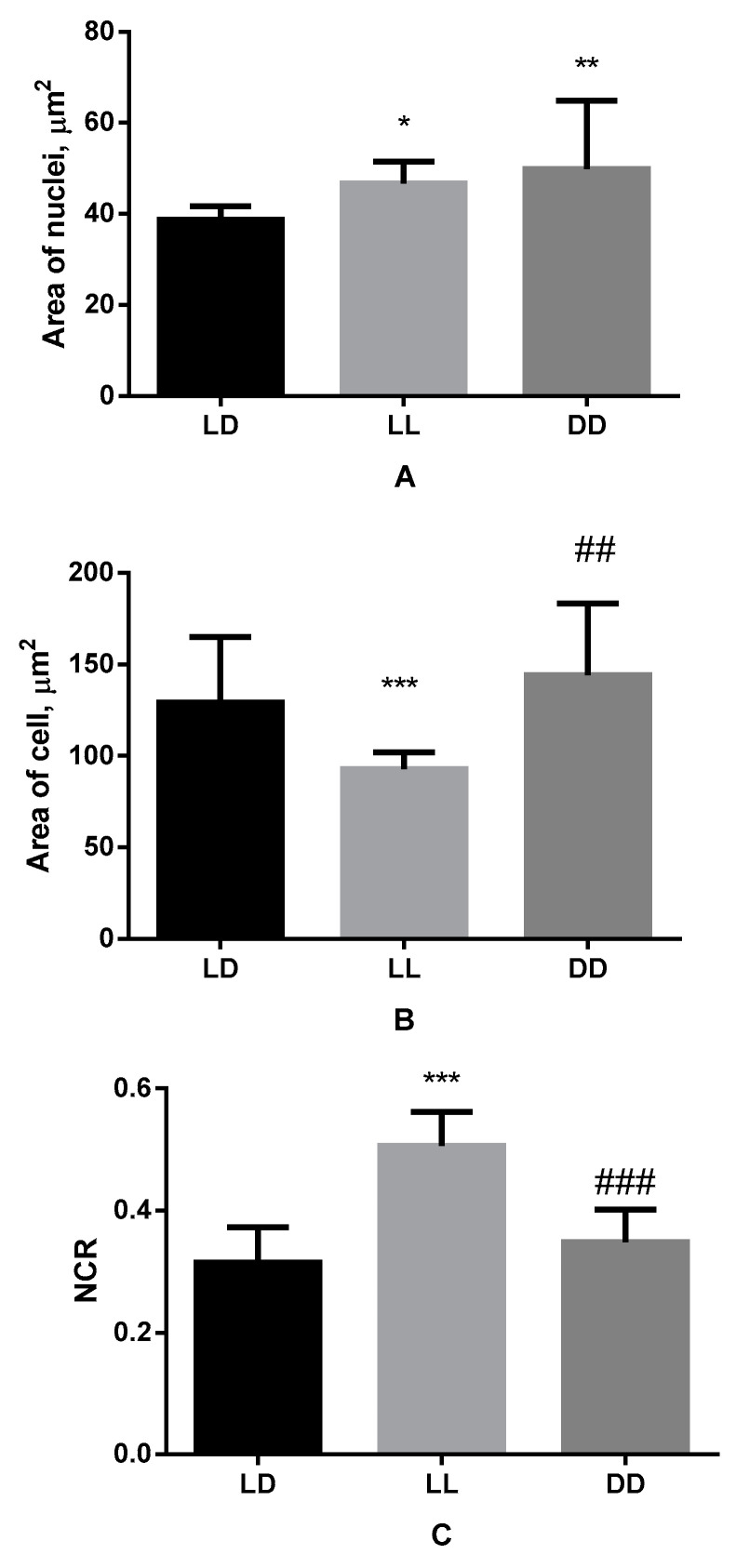
Influence of various lighting regimes on cell nuclei area (**A**), cell area (**B**) and nuclear-cytoplasmic ratio (**C**) of cells of B16 melanoma. * (*p* ≤ 0.05); ** (*p* ≤ 0.005), *** (*p* ≤ 0.0005);—differences in comparison with the parameters of control group mice; ## (*p* ≤ 0.005); ### (*p* ≤ 0.0005)—differences in comparison with mice of LL group.

In particular, area of nuclei of melanoma, which was 38.74 ± 0.67 μm^2^ in control group, increased up to 46.71 ± 4.79 μm^2^ under the influence of constant lighting and up to 49.93 ± 14.87 μm^2^ in conditions of constant darkness (Figure 3).

At the same time the size of the cell itself changed reliably in comparison with control value (which was 129.20 ± 35.87 μm^2^) just in case of constant illumination conditions, making up 92.76 ± 9.24 μm^2^, which is also lower than the same parameter in condition of constant darkness (144.30 ± 38.98 μm^2^) (Figure 3).

The same pattern, accordingly, was noted in relation to the NCR, which amounted 0.32 ± 0.058 in the control group, increased at constant lighting up to 0.51 ± 0.050, and was equal to 0.34 ± 0.051 in the melanoma cells of animals kept in constant darkness (Figure 3).

### 3.4. Serum Melatonin Concentration

**Figure 4:**
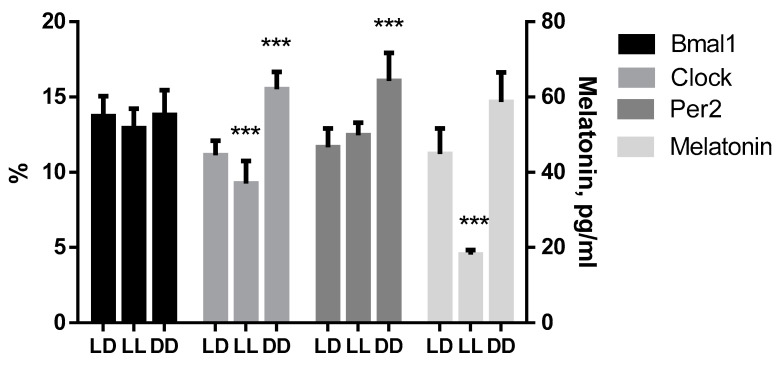
Influence of various light regimes on expression of studied genes. *** (*p* ≤ 0.0005)—differences in comparison with the parameters of control group mice.

Melatonin concentration in blood serum of control animals was 44.88 ± 6.77 pg/mL. In mice kept under constant illumination the same parameter value decreased reliably to 18.16 ± 1.15 pg/mL. In animals of DD group melatonin concentration increased up to 58.70 ± 7.90 pg/mL (Figure 4).

### 3.5. Influence of Various Lighting Regimes on Clock Genes Expression

During the analysis of expression of *Bmal1*, we did not find any intergroup differences. At the same time, *Clock* expression decreased under the constant lighting, amounting to 9.28 ± 1.49% against of 14.15 ± 0.93% in the control, and at constant darkness it increased, reaching 15.52 ± 1.15%. Moreover, under conditions of constant darkness, an increase in the expression of *Per2* was noted (16.08 ± 1.86% against of 11.67 ± 1.24% in the control group) (Figure 4).

## 4. Discussion

The conducted study indicates that the lighting regime has a significant impact on the morphofunctional state of transplantable melanoma. In particular, it was established that animals subjected to darkness deprivation within 2 weeks demonstrate more intensive tumor growth, and keeping of mice in conditions of light deprivation, on the contrary, acts as a tumor growth inhibitor. Furthermore, in tumors of DD group animals the processes of tumor regression were noted, which are also confirmed by the results of micromorphometric studies.

These facts are explained by two effects that occur at staying under specific lighting regime. Firstly, production of pineal melatonin is significantly reduced under conditions of constant lighting [59] which was also confirmed in our study. Secondly, constant light or darkness causes disruption of circadian rhythmicity of an organism [60]. It is known that, in relation to the skin, melatonin can act against damage of DNA and mitochondria and oxidative stress, and prevent inflammation induced by such factors of environment as a stress, solar radiation, poor nutrition or air pollution [61]. Moreover, melatonin has a wide range of antitumor effects, which were noted earlier (immune-potentiating action, antioxidant activity and also inhibitory action on tumor cells growth, angiogenesis and hypoxia-inducible factor-1 (HIF-1)), its antineoplastic efficacy was shown in relation to a number of neoplasms [62,63,64,65].

Our data show that melatonin deficiency leads to pronounced increase mitotic figure numbers in tumors due to the absence of the antiproliferative effect of this hormone [66,67]. In turn, greater mass of the tumor in mice of this group is explained by the fact that cytotoxic effects of melatonin, described for various tumors, do not appear under conditions of its deficiency [68,69]. In turn, in animals kept in conditions of constant darkness, a significant decrease in mitotic activity and consequent slowdown in tumor growth were noted even in comparison with the control group.

Melatonin plays a significant role in circadian rhythmsity structure of mammalian organism. It is well known that disruption of the daily dynamics of melatonin production caused by light/darkness cycle violations, as well as a decrease in its production as a result of exposure of the organism to constant light conditions, leads to disruption of the normal circadian rhythm of an organism. This, in turn, is the basis for the development of a number of pathologies, including oncological ones [70,71].

As for the internal organs of humans and animals, the presence of circadian control of functions has been shown for the skin. However, a number of studies showed that the main clock genes (*Bmal1*, *Clock*, the *Per* family, *Cry* and others) function in various skin cells, and their work can be modulated by a number of additional factors (external light, UV light, feeding/insulin, sleep/insomnia etc.) [72,73,74].

It is established that all cells of melanoma have their own pronounced independent circadian rhythms with different characteristics of oscillation frequency and amplitudes of expression shifts of clock genes [75]. Regardless of whether this dysregulation of clock genes system is one of the causes of skin tumorogenesis or just one of its symptoms, changes in the expression of the clock genes studied by us indicate that constant illumination or darkness causes a disturbance in the chronostructure of tumor cells.

Within the conducted study, we found that there was a change in the expression number of the main clock genes in tumor parenchyma cells as a result of staying in conditions of constant illumination and constant darkness. It is shown that constant lighting leads to the suppression and significant disruption of clock genes expression, which, in accordance with the literature data, aggravates the severance of pathology; on the contrary, constant darkness promotes clock genes expression which creates more favorable conditions for maintaining circadian homeostasis; the presence of synchronization of the circadian characteristics of the tumor and the host organism contributes to the implementation of the antitumor response [76,77,78]. Wherein, melatonin deficiency caused by keeping animals under constant illumination led to more significant tumor growth compared to the control; and, vice versa, keeping animals in the dark not only inhibits this process, but also leads to tumor regression, which is as well reflected in the corresponding fluctuations of mitotic activity of melanoma cells.

It remains an open question whether changes in clock genes expression are a consequence or one of the causes of tumorogenesis, as well as how the violation of the lighting regime affects the circadian rhythm of the studied tumor parameters, which is a goal for further research.

## Data Availability

The data presented in this study are available within the article text, tables, and figures.
